# Gas Chromatography–Sensor System Aids Diagnosis of Inflammatory Bowel Disease, and Separates Crohn’s from Ulcerative Colitis, in Children

**DOI:** 10.3390/s24155079

**Published:** 2024-08-05

**Authors:** Rachael Slater, Kukatharmini Tharmaratnam, Salma Belnour, Marcus Karl-Heinz Auth, Rafeeq Muhammed, Christine Spray, Duolao Wang, Ben de Lacy Costello, Marta García-Fiñana, Stephen Allen, Chris Probert

**Affiliations:** 1 Department of Molecular & Clinical Cancer Medicine, Institute of Systems Molecular and Integrative Biology, University of Liverpool, Liverpool L69 3GE, UK; rsh14@liverpool.ac.uk; 2 Department of Health Data Science, Institute of Population Health, University of Liverpool, Liverpool L69 3GF, UK; k.tharmaratnam@liverpool.ac.uk (K.T.); martaf@liverpool.ac.uk (M.G.-F.); 3 Faculty of Health and Life Sciences, University of Liverpool, Liverpool L69 7ZB, UK; s.belnour@liverpool.ac.uk; 4 Paediatric Gastroenterology, Alder Hey Children’s NHS Foundation Trust, Liverpool L12 2AP, UK; marcus.auth@alderhey.nhs.uk (M.K.-H.A.); stephen.allen@lstmed.ac.uk (S.A.); 5 Gastroenterology and Nutrition, Birmingham Children’s NHS Foundation Trust, Birmingham B4 6NH, UK; rafeeq.muhammed@nhs.net; 6 Paediatric Gastroenterology, Bristol Children’s NHS Foundation Trust, Bristol BS2 8BJ, UK; christine.spray@uhbw.nhs.uk; 7 Department of Clinical Sciences, Liverpool School of Tropical Medicine, Liverpool L3 5QA, UK; duolao.wang@lstmed.ac.uk; 8 School of Applied Sciences, University of the West of England, Bristol BS16 1QY, UK; ben.delacycostello@uwe.ac.uk

**Keywords:** volatile organic compounds, biomarkers, paediatric inflammatory bowel disease, stool, gas-chromatography

## Abstract

The diagnosis of inflammatory bowel disease (IBD) in children and the need to distinguish between subtypes (Crohn’s disease (CD) and ulcerative colitis (UC)) requires lengthy investigative and invasive procedures. Non-invasive, rapid, and cost-effective tests to support these diagnoses are needed. Faecal volatile organic compounds (VOCs) are distinctive in IBD. VOC profiles can be rapidly determined using a gas chromatography–sensor device (OdoReader©). In an inception-cohort of children presenting with suspected IBD, we directly compared the diagnostic fidelity of faecal calprotectin (FCP, a non-specific protein marker of intestinal inflammation) with OdoReader© VOC profiles of children subsequently diagnosed with IBD with matched controls diagnosed with other gastrointestinal conditions. The OdoReader© was 82% (95% confidence interval 75–89%) sensitive and 71% (61–80%) specific but did not outperform FCP (sensitivity 93% (77–99%) and specificity 86% (67–96%); 250 µg/g FCP cut off) in the diagnosis of IBD from other gastrointestinal conditions when validated in a separate sample from the same cohort. However, unlike FCP and better than other similar technologies, the OdoReader© could distinguish paediatric CD from UC (up to 88% (82–93%) sensitivity and 80% (71–89%) specificity in the validation set) and justifies further validation in larger studies. A non-invasive test based on VOCs could help streamline and limit invasive investigations in children.

## 1. Introduction

Inflammatory bowel disease (IBD) is an incurable, chronic, relapsing condition of unknown aetiology. Paediatric Crohn’s disease (CD) tends to be more extensive than CD in adults, with greater involvement of the upper intestine. In addition, CD affecting only the colon is more common in children than in adults, making the distinction between CD and ulcerative colitis (UC) a challenge in some patients [1]. Colonic inflammation in children with UC also tends to be more extensive than in adults [2].

At initial presentation in children with suspected inflammatory bowel disease (IBD), investigations are informed by internationally agreed guidelines and include upper and lower intestinal endoscopy and biopsy (requiring two days of bowel cleansing and usually a general anaesthetic) and small bowel imaging by magnetic resonance enterography or wireless capsule endoscopy [3]. Although serious adverse events are uncommon, these relatively invasive investigations may be stressful for children and their families and consume considerable hospital resources. Undertaking these investigations may delay diagnosis and the start of treatment.

Accurate, non-invasive, rapid, and cost-effective diagnostic tests for IBD that can be used in the clinic or ward setting are needed. Faecal calprotectin (FCP), a marker of intestinal inflammation, is recommended by the National Institute for Health and Care Excellence to help to distinguish IBD from non-inflammatory gut disorders such as irritable bowel syndrome (IBS) [4]. A meta-analysis reported a pooled sensitivity and specificity of FCP in the diagnosis of IBD of 0.98 (95% confidence interval: 0.95–1.0) and 0.68 (0.50–0.86), respectively [5]. The low specificity limits the ability to distinguish IBD from other causes of intestinal inflammation. Furthermore, FCP does not distinguish between CD and UC; tests that can differentiate CD from UC and determine the region of bowel affected would help inform clinical management.

There is great interest in the characterisation of volatile organic compounds (VOCs) in the diagnosis and monitoring of a wide range of diseases. VOCs emitted from stool are responsible for stool odour and consist of a large number of carbon-based molecules of low molecular mass (<1.5 Kd), including organic acids, alcohols, esters, heterocyclic compounds, aldehydes, ketones, and alkanes. VOCs result mainly from the metabolism of the intestinal mucosa and the gut microbiota and their abundance changes according to the specific effects of intestinal diseases on these processes [6].

In adults, a recent meta-analysis of 10 studies characterising VOCs consisted of 696 IBD cases and 605 controls and reached a pooled sensitivity and specificity of 87% and 83%, respectively [7]. This demonstrates the potential for VOC analysis to assist in IBD diagnosis. In children, metabolic profiling for the diagnosis of IBD, differentiation of subtype, and prediction of response to treatment gathering pace [8,9], with a number of studies evaluating the diagnostic potential of faecal VOCs [10,11,12,13].

We have developed a GC–sensor system (OdoReader©) consisting of a GC column in combination with a metal oxide gas sensor for the analysis of VOCs using advanced statistical methods [14]. The OdoReader© is a university-built prototype device for the analysis of headspace gases from biological samples. The gas sensor detector technology was shown to be equivalent in sensitivity (limit of detection) to a mass spectrometer for the detection of a broad range of VOC standards and relevant biological samples (e.g., stool and bacterial cultures) [15]. Currently, we are conducting clinical studies to determine its potential for use in a range of clinical applications, such as faecal analysis in patients with gastrointestinal disorders. The OdoReader© was 78% accurate in differentiating adults with active IBD (n = 83) from symptom-free controls (n = 41) and 92% accurate for IBS (n = 28, defined by Rome II criteria) compared to active IBD [14] using internal validation. We plan to integrate software that will interpret the sensor output in real-time, so that the device could be used at point of care and operated by clinical (but non-scientific) staff to give rapid diagnoses in the clinic or on the ward. This technology has not previously been tested in paediatric IBD.

We characterised faecal VOCs by GC–MS in an inception cohort of children presenting to specialist gastroenterology services with suspected IBD [16]. The primary objectives were to test whether the OdoReader© faecal VOC profiles could distinguish IBD from other gastrointestinal disorders in a training cohort, and then validate the models in a separate sample from the same recruited cohort. Secondary objectives were to evaluate the ability of the OdoReader© to differentiate between disease subtype (CD and UC). Finally, we aimed to determine if baseline faecal OdoReader© profiles could be distinguished from 3-month follow-up profiles of children who had a response to treatment.

## 2. Materials and Methods

### 2.1. Patient Screening and Recruitment

This study is nested within a previous GC–MS study [16]. Patient screening, recruitment, and stool sample collection were performed as previously detailed [16]. In brief, children with suspected IBD that were attending paediatric gastroenterology clinics in 3 UK children’s hospitals (Alder Hey Children’s Hospital, Liverpool; Bristol Royal Hospital for Children, Bristol; and Birmingham Children’s Hospital, Birmingham) were recruited between June 2017 and June 2020.

Following review of referral information, the parents/guardians of children with suspected IBD were sent written guidance requesting collection of a faecal sample the day before or the morning of the clinic visit. They were asked to collect 2–3 scoops of stool using a FecesCatcher (Alpha Laboratories, Eastleigh, UK) and spatula, or an equivalent volume of liquid stool, into a hard plastic Sterilin tube. If collected the day before clinic, the tube, double-bagged in zip-lock plastic bags, was stored in the home freezer and then brought to the clinic at ambient temperature. A stool sample was collected from children referred directly to the ward. The samples were stored at −80 °C until analysis.

Following clinical review, children were excluded if they had already started treatment for IBD or had been diagnosed with another significant intestinal disorder. A diagnosis of IBD and classification of disease sub-type was based on standard clinical guidelines including gastrointestinal endoscopy and biopsy and imaging. IBD was classified according to the Paris classification [3]. Disease severity in CD was defined according to the weighted Pediatric Crohn’s Disease Activity Index (wPCDAI), where a score of ≤12.5 in the absence of corticosteroid treatment, 12.5–40, >40–57.5, and >57.5 indicated clinical remission, mild, moderate, and severe disease, respectively [17]. Disease severity in UC was according to the Pediatric Ulcerative Colitis Activity Index (PUCAI), where <10 in the absence of corticosteroid treatment, 10–34, 35–64, and 65 or above indicated clinical remission, mild, moderate, and severe disease, respectively [17]. The children with IBD were treated following established clinical guidelines [18,19,20]. At 3 months, disease activity was re-assessed as described above, and a further stool sample was requested for analysis using the GC–sensor. The stool samples were collected and stored as baseline samples. A decrease in disease activity category was regarded as a response to treatment.

The diagnosis of non-IBD gastrointestinal disorders followed usual clinical practice. Children in whom IBD was diagnosed were matched with one non-IBD child with other gastrointestinal conditions for age (+/−6 months), sex, and recruitment site [10].

FCP was measured in each hospital as follows. Bristol Children Hospital used the ELISA Bühlmann fCAL Calprotectin kit for DS2 (normal value < 50 µg/g) (BÜHLMANN Laboratories AG, Schönenbuch, Switzerland). Alder Hey Children Hospital used the same method until September 2018, and then an EliA Calprotectin fluorescence enzyme immunoassay was used for the remainder for the study (normal value < 50 mg/kg). Birmingham Children Hospital used Bühlmann Calprotectin ELISA kit (EK-CAL; normal value < 60 µg/g).

A total of 152 baseline samples and 37 IBD 3-month follow-up stool samples were available from the GC–MS study. From these, a subset of IBD-matched to non-IBD pairs were selected at random for this proof of principle study. In this exploratory analysis, we analysed a greater number of samples than the Aggio study in adults, which reported 19 cases of active CD, 14 cases of active UC, and 41 controls [14].

### 2.2. Analysis of Stool Samples by GC–Sensor

The samples were analysed using a GC–Sensor (OdoReader©) device, as described previously [14]. Aliquots of stool (450 mg in 10 mL headspace vials) were defrosted at room temperature for 15 min before being heated for 10 min at 50 °C. The 2 mL headspace gas was then injected onto a 30 m SPB, 1 sulphur GC column (Supelco; Sigma-Aldrich, St. Louis, MO, USA) of the OdoReader© for separation, facilitated by a synthetic air carrier gas (BOC, Guildford, UK). The specific GC temperature was held at 40 °C for the first 3.42 min) then ramped at a rate of 2.5 °C per min to 100 °C. During the 40 min total runtime, VOCs eluting from the column then reached a bespoke metal oxide sensor. The properties of the metal oxide sensor have been described in detail previously [15]. The temperature of the metal oxide sensor (450 °C) was controlled by an electronic circuit monitored by a computer system. The electronic resistance of the sensor was recorded at 0.5 s intervals during the run, and the resistance profile for each sample was stored in a text file format. The samples were run in a randomised order, and the laboratory staff were blinded to the patient’s diagnosis and response to treatment in those with IBD.

Samples were run in two batches. The first sample set consisted of 48 IBD stools (26 CD, 18 UC and 4 IBD unclassified (IBDU)), 48 matched controls, and 23 IBD 3-month follow-ups and was run between September 2019 and January 2020. A second set of validation samples (28 IBD (16 CD, 10 UC, 2 IBDU each matched with a control) and 14 IBD 3-month follow-ups) from the same recruited cohort were run later (August–September 2020) following a laboratory shutdown during the COVID-19 pandemic and a change of metal oxide sensor. The properties of the metal oxide sensor remained the same as the one used in the training set, and all other instrumental and methodological parameters were the same.

### 2.3. Statistical Analysis

An in-house-developed computer pipeline was used to analyse the OdoReader© chromatogram resistance profiles, as reported previously [21]. In summary, the OdoReader©-generated files were loaded into R (version 3.1.1), and baseline correction, normalisation (based on highest resistance within a sample), alignment, and transformation based on wavelet coefficients were performed as described previously [21]. To account for baseline differences between batches, all samples including the training and validation cohorts were initially processed together. At this stage the validation cohort was then held back for later testing. From this point, in addition to the in-house-developed pipeline, the least absolute shrinkage and selection operator (LASSO) approach introduced by Tibshirani [22] was also applied as an alternative model for feature selection and classification of groups. Both statistical approaches are described below.

For the pipeline described previously, feature reduction (to 100 features from 3601) was initially achieved using univariate tests [21]. A feature selection process was then followed, based on random forest using the combined selected features from two algorithms: boruta [23] and recursive feature selection (RFE) [24]. A total of 7 different classifiers (k-nearest neighbour (KNN), partial least squares (PLS), random forest (RF), linear discriminant analysis (LDA), support vector machine (SVM) with radial basis function kernel (SVMR), SVM with linear basis function kernel (SVML), and SVM with polynomial basis function kernel (SVMP) were then applied to classify the sample groups. For the second pipeline, feature selection was conducted using LASSO [22]. By removing irrelevant variables, the LASSO approach [22] tends to increase the prediction accuracy and interpretability when the ratio of the sample size and the number of variables is relatively small. The LASSO approach has two steps. First, a LASSO logistic regression model is used to select the most important features; second, a logistic regression model is fitted using the selected features, and the model is subsequently used in sample classification. The *glmnet* package in statistical software R was used to run the procedure.

For both pipelines, the models were built using the training datasets, and these models (which include the selected features) were tested in the validation sets. Principal component analysis (PCA) plots were generated using the selected features from each model to visualise group separation. Boxplots of the selected features were also generated. For further validation, the analysis pipeline [21] utilised three different cross validation techniques (cross validation (CV), double cross validation (DCV), and leave one out cross validation (LOOCV)). The sensitivity and specificity values are selected with respect to the optimal threshold (maximum of (sensitivity + specificity)) from the area under the curve (AUC).

### 2.4. Faecal Calprotectin

We calculated the sensitivity and specificity of FCP in the diagnosis of IBD using two different cut-offs: 100 µg/g [4] and 250 µg/g [25].

## 3. Results

### 3.1. Patient Characteristics

**Figure 1:**
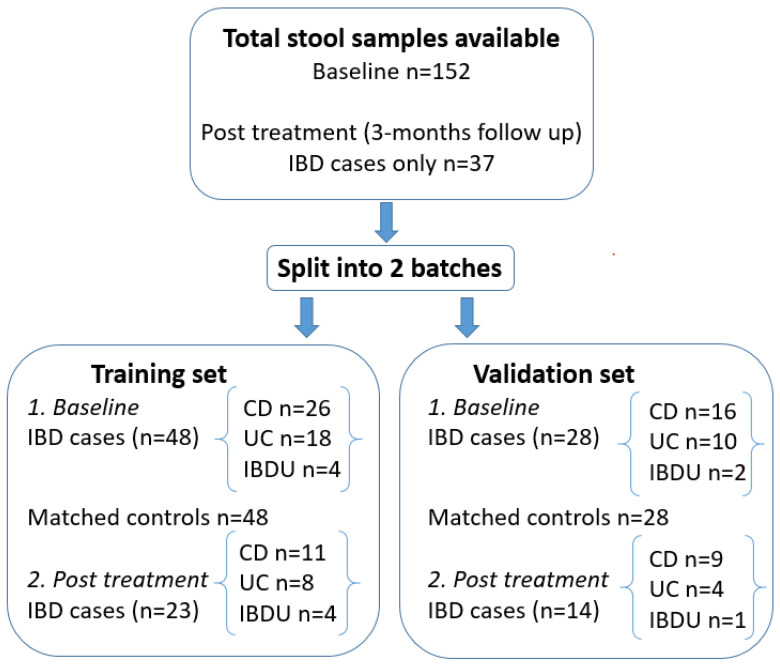
A flow chart summary of stool samples analysed from children with IBD and non-IBD-matched controls.

A flow diagram summarising the patient sample numbers included in this study is shown in Figure 1. Similar distributions for age and sex were observed between groups within and between the training and validation datasets (Table 1). In the non-IBD-matched controls for both datasets, the most common diagnoses were no evidence of a gastrointestinal disorder, followed by functional abdominal pain not otherwise specified (FAP-NOS), and IBS (Supplementary Table S1).

All IBD cases at the 3-month follow-up were either in remission or had mild disease activity (Table 1). The age, sex, and 3-month follow-up disease activity scores for these patients are summarised in Table 1, and the treatment regimens that were included in the analysis are shown in Supplementary Table S2.

### 3.2. GC–Sensor Modelling

**Figure 2:**
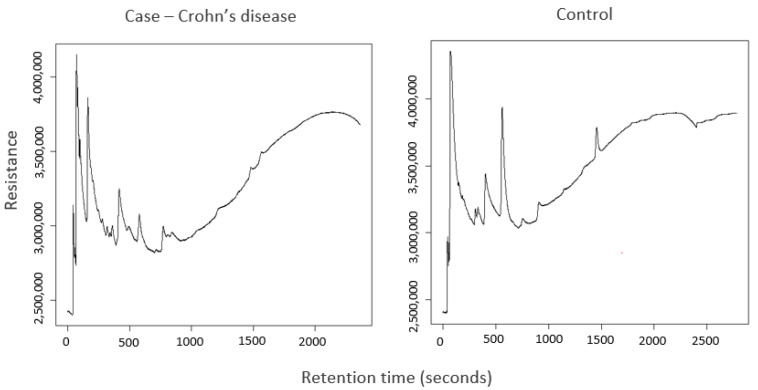
OdoReader©-generated chromatogram profiles of analysed stool from a child with Crohn’s disease and a child with functional constipation.

The performance of each classifier model is dependent on the group comparisons being made. Further details of the classifiers used and the scheme for cross-validation using this pipeline have been described previously [21]. However, in general, the SVML classifier with LOOCV for validation is the overall best performing model in the validation datasets to generate values for accuracy, sensitivity, and specificity for all comparisons. We have chosen to report one model across all comparisons rather than the best model for each comparison to avoid bias. A list of group comparisons is summarised in Supplementary Table S3. The results for all training models are reported in Supplementary Tables S4–S7, and all validation models are reported in Supplementary Tables S8–S11. An example of the OdoReader© chromatogram profiles is shown in Figure 2 for a case (Crohn’s disease and a control (functional constipation).

#### 3.2.1. IBD vs. Non-IBD Controls

**Figure 3:**
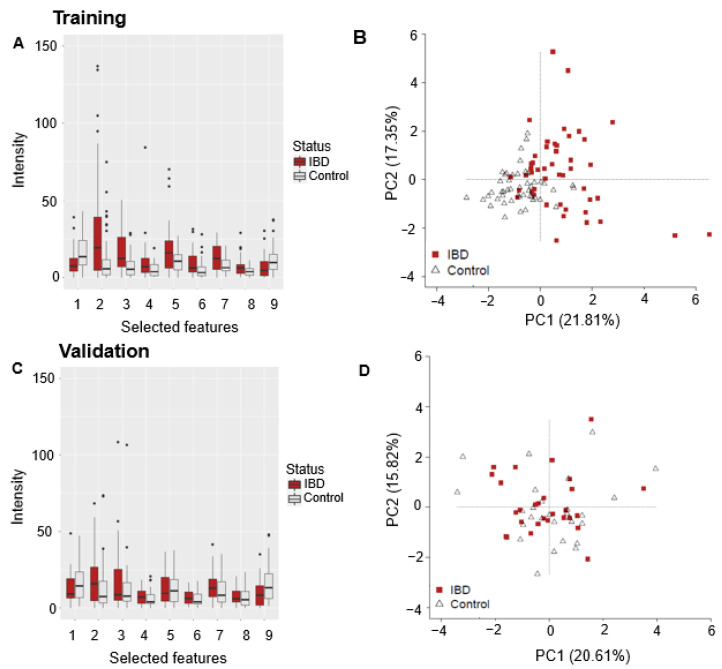
IBD vs. controls training and validation data. Boxplots of features selected by the model in the training set (**A**) and the same features used in the validation (**C**). PCAs based on selected features for both training (**B**) and validation sets (**D**).

In IBD (combining CD, UC, and IBDU) compared to matched controls, the trends in feature abundance were similar across the training and validation sets (Figure 3A,C). A total of 7 out of 9 selected features were, on average, more abundant in IBD than controls in the training set (Figure 3A). In the validation set, six out of seven features remained more abundant in IBD, and the two that were more abundant in the controls remained so in the validation set (Figure 3C). Separation between IBD and controls was not observed in the PCA (Figure 3D) for the validation set, despite an accuracy of 75% (95% confidence interval (CI 70–80%) with 82% sensitivity (75–89%) and 71% specificity (61–80%).

#### 3.2.2. CD/UC vs. Controls

The next comparison treated CD and UC separately. The selected features used to separate CD from matched controls are shown in Supplementary Figure S1. The validated model had an accuracy of 75% (CI 69–81%) and a sensitivity and specificity of 83% (74–92%) and 70% (60–80%), respectively. Supplementary Figure S2 shows the selected features for the separation of UC and matched controls, and the validated model had an accuracy of 50% (38–62%), sensitivity of 50% (32–68%), and specificity of 50% (32–68%). When adding the IBDU cases to the UC group, the new model (Supplementary Figure S3) achieved an improved accuracy of 75% (65–86%), sensitivity of 71% (52–91%), and specificity of 80% (64–96%) when comparing IBDU/UC with their matched controls.

#### 3.2.3. CD vs. UC

**Figure 4:**
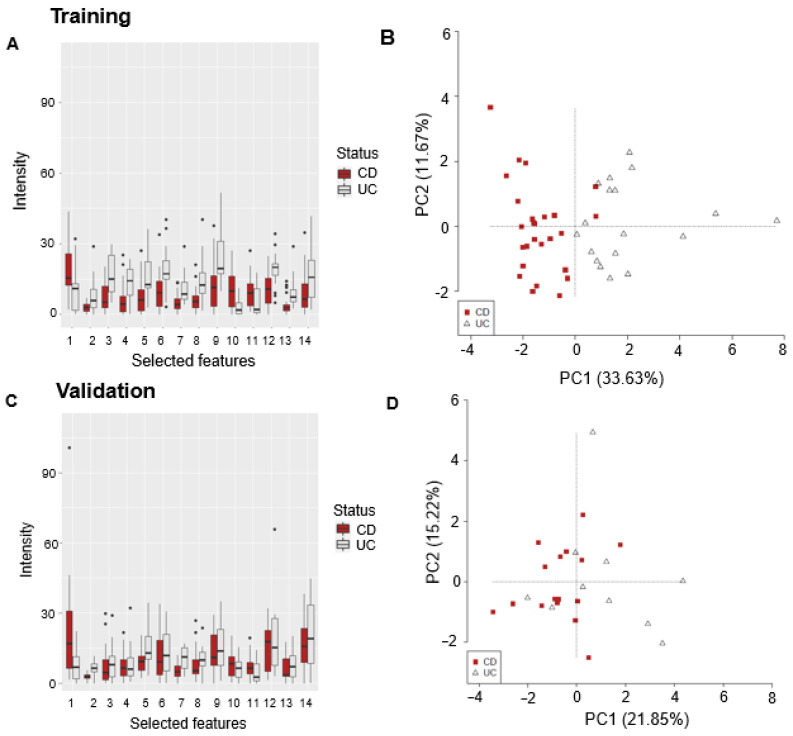
CD vs. UC training and validation data. Boxplots of features selected by the model in the training set (**A**) and the same features used in the validation (**C**). PCAs based on selected features for both training (**B**) and validation sets (**D**).

A further model was built to determine whether the GC–sensor VOC profiles could distinguish between CD and UC. A total of 14 features were selected by the training model to distinguish between CD and UC (Figure 4A,C), and 12 out of 14 features showed, on average, significantly different levels of abundance when comparing CD and UC groups, with consistent trends (directions) in the training and validation sets. The SVML followed by LOOCV had an accuracy of 73% (CI 69–77%) (80% sensitivity (75–85%)), 64% specificity (54–74%)) when distinguishing between CD and UC. The PCAs illustrating the separation of CD and UC based on model-selected features are shown in Figure 4B,D for the training and validation sets, respectively.

#### 3.2.4. Baseline Active vs. 3-Month Follow Up Remission

To investigate whether the GC–sensor VOC profiles are altered post-treatment in children who achieved clinical remission, a subset of the baseline IBD samples were compared to the 3-month follow-ups of the same children (23 pairs included in the training set, 14 pairs included in the validation). A total of 13 features were selected to differentiate between baseline IBD cases and their 3-month follow-up samples (Supplementary Figure S4). Six features were consistently higher at baseline than follow-up in the training and validation sample sets. Five features were consistently higher at follow-up in both the training and validation sample sets (Supplementary Figure S4). Despite a largely consistent change in features in the two datasets, separation for baseline and follow-up was not observed in the PCA when tested on the validation set (Supplementary Figure S4).

#### 3.2.5. LASSO Modelling

The LASSO models were fitted (Table S7) and then tested in the validation cohort (Table S11). Overall, the models were less accurate than the models developed using the first pipeline [21], with the most accurate Lasso model (68% CI 54–82%) being achieved to separate UC from matched controls with a sensitivity of 80% (68–92%) and a specificity of 50% (28–72%).

### 3.3. Faecal Calprotectin

Using the 100 µg/g FCP cut-off for the diagnosis of IBD, the test was 96% (CI 86–99%) sensitive, 81% (67–91%) specific in the training cohort; and 96% (82–100%) sensitive and 82% (63–94%) specific in the validation cohort. The accuracy for the training cohort was 89% (80–94%) and 90% (78–96%) for the validation cohort. When the 250 µg/g cut-off was applied, the FCP was 92% (80–98%) and 93% (77–99%) sensitive, 96% (86–99%) and 86% (67–96%) specific, with accuracies of 94% (87–98%) and 90% (78–96%) for distinguishing between IBD cases and controls in the training and validation cohorts, respectively. A comparison of the validation cohort of the OdoReader© and FCP performance in distinguishing between cases and controls is shown in Table 2.

## 4. Discussion

This is the first prospective study of the OdoReader© in paediatric IBD. Few studies have tested the models on a separate validation set in adults. All the patients were newly presenting with gastrointestinal symptoms suggestive of IBD and underwent comprehensive investigation by paediatricians in tertiary centres. The data show the potential utility of faecal gas analysis in the evaluation of children with a potential diagnosis of IBD. Although it is common to see, like in this study, that separation is clearly better in the training than the validation data, the latter gives a more realistic assessment of this technology. The accuracy in defining a heterogeneous group of IBD patients (including CD, UC, and IBD-U) was 75%; CD and UC could be distinguished from non-IBD controls with other gastrointestinal conditions with accuracies of 75% and 50%, respectively, and from each other with an accuracy 73%. To our knowledge, this is the best performing non-invasive model for separating CD and UC when validated with a separate sample set.

Previously, the OdoReader© device was able to separate IBS from IBD in an adult cohort with high accuracy (92%) after internal double cross-validation [14]. Field asymmetric ion mobility spectrometry (FAIMS) differentiated between IBD with a FAP-NOS/IBS group in a paediatric cohort with high accuracy (94%) [10]. Others, using FAIMS or GC–ion mobility spectrometry (GC–IMS) have reported sensitivities and specificities of 79–97% and 78–97%, respectively, when comparing IBD with healthy controls [10,13,26]. However, comparisons with healthy controls do not represent the real-life clinical application. Comparison of IBD with functional and other GI disorders has merit for investigating the potential of faecal VOC-pattern technologies for assisting in the diagnosis of IBD in routine clinical practice.

We recruited children suspected of IBD but with a broad range of GI disorders from gastroenterology clinics at three different sites. Two other studies using GC–IMS compared the stool of children with IBD and controls with a broad range of GI disorders [11,12]. The smaller study of the two consisting of 20 children reported an AUC of 0.73, a sensitivity of 70%, and a specificity of 90% [12]. The larger study consisting of a case–control study of 109 IBD cases and 75 controls with a broad range of GI disorders demonstrated significant differences in VOC profiles but could not separate IBD cases from controls with high accuracy after internal validation (AUC of 0.71, sensitivity of 59%, specificity of 77%) [11]. The results from both works are comparable to the current study (accuracy of 75%, a sensitivity of 82%, and a specificity of 71%), which we validated in a separate sample set recruited from the same cohort. These findings are similar to those comparing IBD to functional GI disorders and healthy controls, indicating that faecal VOC-pattern technologies could be of use for assisting the diagnosis of IBD in a clinical setting.

In this study, children with IBD were matched for age, sex, and recruitment hospital to non-IBD controls to account for differences in the faecal VOC profile that may have been as a consequence of these factors. Other potential environmental confounding factors, e.g., diet, other medications, and antibiotic exposure were not controlled for in the current study as we aimed to identify biomarkers for IBD in newly presenting cases which would reflect the real-life clinic. However, using an electronic nose to study the faecal VOC profiles of healthy subjects, body mass index and many environmental factors, including diet and medications, were significant factors in influencing the VOC profile [27]. The authors recommend the inclusion of such factors when developing biomarker models.

Separation of CD from non-IBD controls with other gastrointestinal conditions generated one of the best models in this study (accuracy 75%, sensitivity 83%, specificity 70%), as similarly found by others comparing CD, UC, and IBD to healthy controls [13]. However, in this paediatric cohort, the OdoReader© was not able to separate UC from controls with high accuracy. The addition of six IBDU cases and their matched controls across the training and validation sets seemed to improve accuracy and class predictions, which may indicate that the UC vs. controls comparison was underpowered. We observed that small changes in numbers seem to make a substantial difference in the results, which highlights the need to externally validate the models. Previous FAIMS studies have reported sensitivity of 77% and specificity of 75% for UC vs. HC and a sensitivity of 100% and specificity of 80% for UC vs. FAP-NOS/IBS controls in paediatric cohorts [10,13]. The separation of active UC from other GI disease controls may be more challenging than for CD using faecal VOC-pattern technologies. However, this requires further investigation in larger sample sizes, as to the best of our knowledge, our study is the first to make this comparison.

In terms of differentiating between CD and UC, the results of our SVLM with LOOCV model were comparable to previous studies reporting sensitivities of 60–65% [10] and specificities of 62–80% [13] in paediatric cohorts. In an adult cohort [14], after internal double cross-validation, CD was separated from UC with a sensitivity of 99% and a specificity of 93%. The adult cohort in the latter study may have observed a drop in performance if an external validation group had been used to test the model. In a GC–IMS cohort of adult IBD cases (n = 280) and healthy controls (n = 277), reserving a subset to test their models [26], poorer class predictions (a sensitivity 74% of and a specificity of 43%) for active CD and UC were observed. We have chosen to focus on one type of model (SVML) in our analysis; however, the model using LDA performed better on this occasion with 88% sensitivity and 80% specificity. More work is required to select the optimum modelling approach as the optimal method chosen may not be the same if a new dataset is used.

Despite some decrease in disease activity, the VOC profiles between baseline and 3-month follow-up were not distinguishable in the PCA for the validation set. The VOC profile remained similar to pre-treatment, potentially as a result of persistent dysbiosis, but microbiota profiling would be required to confirm this. Some patients had mild disease activity at follow-up, so it may be possible that the decrease in disease activity was not enough to observe changes. Furthermore, these results also suggest the VOC patterns observed in this study may not simply be markers of inflammation, as the patterns remain similar at follow-up when the majority of FCP values have decreased to below levels that indicate inflammation. Faecal VOC patterns may have the potential to complement other diagnostic techniques, including FCP, and to differentiate between IBD sub-types but may not have the potential to monitor disease activity, consistent with similar results reported in adult IBD cohorts [14,26].

In this cohort, the OdoReader©’s performance in defining cases of IBD was less accurate than FCP (75% vs. 90%, respectively). However, the potential for the OdoReader© to separate CD from UC performed better than other similar technologies where a separate sample from the same cohort had been used to test the model. The 250 ug/g cut-off for FCP was marginally more accurate than 100 ug/g, supporting the work by Orfei and colleagues [25]. Furthermore, other stool protein markers have previously proved unsuccessful in distinguishing between CD and UC [28]. GC–sensor-based technology may offer an alternative non-invasive solution to assist IBD subtype diagnosis in children.

Although the OdoReader© technology is not able to determine specific VOCs that underlie potential diagnosis and pathogenesis, the patient samples in this study were part of a larger subset analysed previously using a gas chromatography mass spectrometry system [16]. The diagnostic potentials of individual VOCs were not evaluated by Belnour and colleagues, but propan-1-ol and phenol were increased in pre-treatment IBD cases compared to non-IBD. Abundances of some ketones and fatty acids were increased in CD compared to UC and could, in part, explain the biological differences underlying the differentiation of groups by the OdoReader© in the current study.

A main limitation of this study was the small sample size, particularly for the validation sample, which was smaller than the training set [29]. Further validation in larger cohorts is needed to evaluate the use of the OdoReader© and models for use as an assisted diagnostic tool for paediatric IBD. Larger sample sizes would allow data splits in three sets (e.g., for training, validation, and testing). The sample size did not allow comparisons to be made to determine whether faecal VOC-patterns would be useful for determining the bowel region affected in IBD cases, which would be a useful tool for clinicians. We were not able to exclude children who had taken antibiotics, as this would have restricted the sample size further. Given the relationship of VOCs with the gut microbiome, this may have been a confounding factor [30].

Few VOC-pattern detector studies for IBD biomarkers have previously attempted to validate training models using an external sample set [26], and, to the authors’ knowledge, samples analysed in a separate batch have not been used as external validation of the same models before. The training models performed well in separating suspected IBD cases into confirmed IBD and non-IBD. However, we found that performance dropped when these models were tested with an external validation set. Lack of reproducibility and variability between studies is a known challenge for the practical application of metabolomics technologies as diagnostic tools [9,31]. Despite these limitations and other confounding factors, our external validation cohort obtained sensitivities of 80–83% and specificities of 64–73% for IBD/CD vs. non-IBD with other gastrointestinal conditions and in separating CD from UC, which demonstrates the potential of GC–sensor devices for assisting in the diagnosis of IBD. In agreement with other studies, class predictions were poorer in distinguishing between active and inactive disease. Improved quality control measures to correct for machine operating batch effects and further investigation and incorporation of the effect of confounding factors in biomarker models is needed.

The OdoReader© technology, tested in paediatric IBD for the first time, distinguished between IBD sub-types. Further testing and validation are required to determine if it may assist with non-invasive diagnosis of IBD, and prediction of response to treatment and relapse could lead to better patient outcomes and cost-effective treatment.

## Figures and Tables

**Table 1:** Summary of baseline patient characteristics. Key: CD = Crohn’s disease, UC Ulcerative colitis, IBDU = Inflammatory bowel disease unclassified, disease location defined by the Paris classification: L1: distal 1/3 ileum with or without limited cecal disease; L2: colonic disease; L3: ileocolonic disease; E1: ulcerative proctitis; E2 = left-sided colonic inflammation (distal to splenic flexure); E3: extensive (hepatic flexure distally); E4: pancolitis (proximal to hepatic flexure).

Variable Training Set	CD N = 26	UC N = 18	IBDU N = 4	Non-IBD Controls N = 48	IBD at 3-Months Following Treatment N = 23 (CD = 11, UC = 8, IBDU = 4)
**Age in years (range)**	12.6 (4–16)	11.4 (5–16)	10.5 (8–13)	11.8 (4–16)	11.5 (4–16)
**Female n (%)**	12 (46.2)	8 (44.0)	1 (25)	20 (41.7)	8 (34.8)
**Received antibiotics in last 3 months: no. (%)**	4 (15.4)	0	0	7 (14.6)	-
**Mean (range) disease activity score**	50.7 (12.5–105)	45.0 (20–75)	53.8 (50–60)	-	16.2 (0–40)
**Disease location (%)**	L1 = 7 (26.9) L2 = 6 (23.1) L3 = 13 (50)	E1 = 4 (14.4) E2 = 2 (11.1) E3 = 1 (5.6) E4 = 11 (61.1)	E1 = 1 (25) E4 = 3 (75)	-	-
**Variable** **Validation set**	**CD** **N = 16**	**UC** **N = 10**	**IBDU** **N = 2**	**Non-IBD Controls** **N = 28**	**IBD at 3-Months** **Following Treatment** **N = 14** **(CD = 9, UC = 4, IBDU = 1)**
**Age in years (range)**	10.7 (7–15)	12.7 (8–16)	7–9	10.8 (4–16)	10.9 (7–15)
**Female n (%)**	9 (56.3)	3 (30.0)	1 (50.0)	11 (39.3)	5 (35.7)
**Received antibiotics in last 3 months: no. (%)**	3 (18.8)	1 (10.0)	0	1 (3.6)	-
**Mean (range) disease activity score**	54.2 (25–90)	52 (30–85)	50 (50–50)	-	12.8 (0–35)
**Disease location: no. (%)**	L1 = 5 (31.3) L2 = 5 (31.3) L3 = 6 (37.5)	E1 = 4 (40) E2 = 1 (10) E4 = 5 (50)	E4 = 2	-	-

**Table 2:** Summary of the performance of faecal calprotectin and the OdoReader© GC–sensor device in distinguishing between IBD, controls, and subtypes. All validated model results and accuracies are reported in Tables S8–S11.

	Calprotectin (100 µg/g Cut Off)			Calprotectin (250 µg/g Cut Off)			Sensor Model SVML with LOOCV			Best Sensor Model		
Comparison	Accuracy % (CI)	Sensitivity % (CI)	Specificity % (CI)	Accuracy % (CI)	Sensitivity % (CI)	Specificity % (CI)	Accuracy % (CI)	Sensitivity % (CI)	Specificity % (CI)	Accuracy % (CI)	Sensitivity % (CI)	Specificity % (CI)
**IBD vs.** **control**	90 (78–96)	96 (82–100)	82 (63–94)	90 (78–96)	93 (77–99)	86 (67–96)	75 (70–80)	82 (75–89)	71 (61–80)	75 (70–80)	82 (75–89) *	71 (61–80) *
**CD vs.** **control**	75 (57–89)	94 (70–100)	56 (30–80)	81 (64–93)	88 (62–98)	75 (48–93)	75 (69–81)	83 (75–92)	70 (60–80)	75 (69–81)	83 (75–92) *	70 (60–80) *
**UC vs.** **control**	90 (68–99)	100 (69–100)	80 (44–97)	95 (75–100)	100 (69–100)	90 (56–100)	50 (38–62)	50 (38–62)	50 (32–68)	68 (54- 82)	80 (68–92) †	50 (28–72) †
**UC and IBDU vs.** **control**	92 (73–99)	100 (74–100)	83 (52–98)	96 (79–100)	100 (74–100)	92 (62–100)	75 (65–86)	71 (52–91)	80 (64–96)	75 (65–86)	71 (52–91) *	80 (64–96) *
**CD vs. UC**	*NA*	*NA*	*NA*	*NA*	*NA*	*NA*	73 (69–77)	80 (75–85)	64 (54–74)	85 (79–90)	88 ‡ (82–93)	80 ‡ (71–89)

The pipeline [21] used for the best sensor model for each comparison are * support vector machine with linear basis function kernel (SVML) with leave one out cross validation (LOOCV), † least absolute shrinkage and selection operator (LASSO), and ‡ linear discriminant analysis (LDA) with LOOCV.

## Data Availability

The original data presented in the study are openly available in Mendeley Data: https://data.mendeley.com/datasets/vmwymcjxxw/1 (accessed on 13 June 2024). The R scripts used to perform the analysis are available in the Supplementary Materials.

## Supplementary Materials

The following supporting information can be downloaded at: https://www.mdpi.com/article/10.3390/s24155079/s1. Figure S1: Selected training features and validation for CD vs. Controls; Figure S2: Selected training features and validation for UC vs. Controls; Figure S3: Selected training features and validation for UC/IBDU vs. Controls; Figure S4: Selected training features and validation for baseline IBD vs. 3-month FUP; Table S1: Diagnosis of controls included in the training and validations sets; Table S2: Treatment of 3-month follow-up IBD cases for the training and validation cohorts; Table S3: Summary of comparisons; Table S4: Summary of LOOCV models for training data; Table S5: Summary of CV models for training data; Table S6: Summary of DCV models for training data; Table S7: Summary of LASSO models for training data; Table S8: Summary of LOOCV models for validation data; Table S9: Summary of CV models for validation data; Table S10: Summary of DCV models for validation data; Table S11: Summary of LASSO models for validation data.
