# Supplementary material for: Gas Chromatography–Sensor System Aids Diagnosis of Inflammatory Bowel Disease, and Separates Crohn’s from Ulcerative Colitis, in Children
Source: Sensors (Basel). 2024 Aug 5;24(15):5079. doi: 10.3390/s24155079 (PMC11314755; doi:10.3390/s24155079)
Supplement: Supplementary file 1 [file sensors-24-05079-s001.zip › sensors-3091818-supplementary.pdf]

Table S1 Diagnosis of controls included in the training and validations sets. Key: FAP-NOS = functional abdominal pain - not otherwise specified, IBS = irritable bowel syndrome, ASLC = acute self-limited colitis, CVS = cyclic vomiting syndrome.

Control group	Diagnosis	Number of children (%)
**Training (n=48)**	No diagnosis/normal	12 (25)
	FAP-NOS	11 (22.9)
	IBS	8 (16.7)
	Functional constipation	7 (14.6)
	ASLC	1 (2.1)
	Allergic Enterocolitis	1
	Colonic polyp	1
	CVS	1
	Functional diarrhoea	1
	Gastritis & anal fissure	1
	Non-specific wheat intolerance	1
	Pilonidal sinus	1
	PR bleeding	1
	Rectal polyp	1
**Validation (n=28)**	No diagnosis/other	10 (35.7)
	FAP-NOS	5 (17.9)
	IBS	6 (21.4)
	Functional constipation	3 (10.7)
	Colonic polyp	1 (3.6)
	Coeliac disease	3 (10.7)

Table S2 Treatment of 3-month follow-up IBD cases for the training and validation cohorts

Sample set	Treatment and number of children	Other drugs and number of children
**Training (n=23)**	5-aminosalicylate (Mesalazine) = 6<br>5-aminosalicylate (Mesalazine) and Azathioprine = 2<br>5-aminosalicylate (Mesalazine) and Infliximab = 1<br>Azathioprine = 3<br>Adalimumab = 1<br>Azathioprine and Adalimumab = 2<br>Azathioprine, Steroid (Prednisolone) and Infliximab = 1<br>Dietary supplement (Fortini) and Azathioprine = 1<br>Herbal/alternative medicine (Turmeric milk) = 1<br>Modulen = 1<br>Prednisolone = 2<br>Prednisolone and Infliximab = 1<br>No treatment = 1	Omeprazole = 4<br>Adcal D3 = 1<br>Turbo inhaler = 1<br>Metronidazole = 1<br>Flucloxacillin = 1<br>Movicol = 1<br>CaciChew = 1
**Validation (n=14)**	5-aminosalicylate (Mesalazine) = 4<br>5-aminosalicylate (Mesalazine), Rectal Medication: 5-aminosalicylate (Salofalk) = 1<br>5-aminosalicylate (Sulphasalazine) = 1<br>Azathioprinen = 1<br>Azathioprine and rectal steroid (Prednisolone) = 1<br>Modulen = 1<br>Modulen and Methotrexate = 1<br>Modulen, Methotrexate and Herbal medicine = 1<br>Other dietary supplement (Fortisip exclusive liquid diet) =1<br>Other dietary supplement (Fortisip) and Azathioprine = 1	Laxedo 1-2PRN = 1<br>Omeprazole = 1

Table S3 Summary of comparisons. Key: Inflammatory bowel disease = IBD, Crohn's disease = CD, ulcerative colitis = UC, Inflammatory bowel disease unclassified = IBDU, 3-months follow up = FUP

	Training		Validation	
Comparison	Group1	Group2	Group1	Group2
IBD vs control	IBD (n=48)	Control (n=48)	IBD (n=28)	Control (n=28)
CD vs control	CD (n=26)	Control (n=26)	CD (n=16)	Control (n=16)
UC vs control	UC (n=18)	Control (n=18)	UC (n=10)	Control (n=10)
UC and IBDU vs control	UC and IBDU (n=22)	Control (n=22)	UC and IBDU (n=12)	Control (n=12)
CD vs UC	CD (n=26)	UC (n=18)	CD (n=16)	UC (n=10)
IBD baseline vs IBD FUP	IBD baseline (n=23)	IBD FUP (n=23)	IBD baseline (n=14)	IBD FUP (n=14)

Table S4 Summary of LOOCV models for training data

Comparison	Model	Accuracy (95% CI) (%)	Sensitivity (95% CI) (%)	Specificity (95% CI) (%)
IBD vs control	KNN	80 (78, 82)	82 (79, 86)	78 (76, 81)
IBD vs control	PLS	82 (80, 84)	83 (79, 86)	82 (79, 85)
IBD vs control	RF	82 (80, 85)	80 (77, 84)	84 (81, 88)
IBD vs control	LDA	84 (82, 86)	87 (84, 89)	82 (79, 85)
IBD vs control	SVMR	82 (81, 84)	82 (79, 84)	83 (80, 86)
IBD vs control	SVML	86 (84, 89)	91 (87, 94)	83 (80, 86)
IBD vs control	SVMP	80 (78, 82)	81 (78, 84)	80 (76, 83)
CD vs control	KNN	90 (88, 93)	92 (88, 97)	89 (86, 92)
CD vs control	PLS	92 (90, 95)	92 (88, 97)	92 (89, 95)
CD vs control	RF	92 (90, 94)	92 (89, 96)	92 (89, 95)
CD vs control	LDA	92 (90, 95)	92 (88, 97)	92 (88, 96)
CD vs control	SVMR	92 (90, 94)	87 (85, 89)	100 (97, 100)
CD vs control	SVML	94 (92, 97)	96 (92, 100)	93 (89, 96)
CD vs control	SVMP	94 (92, 96)	93 (90, 95)	96 (93, 99)
UC vs control	KNN	85 (84, 87)	83 (77, 90)	86 (84, 87)
UC vs control	PLS	91 (89, 93)	88 (82, 93)	92 (90, 94)
UC vs control	RF	93 (91, 94)	93 (87, 99)	92 (92, 93)
UC vs control	LDA	88 (86, 90)	78 (73, 83)	92 (90, 94)
UC vs control	SVMR	90 (88, 91)	87 (82, 92)	91 (88, 93)
UC vs control	SVML	88 (86, 90)	78 (73, 83)	92 (90, 94)
UC vs control	SVMP	91 (89, 93)	93 (88, 98)	91 (88, 93)
UC and IBDU vs control	KNN	85 (83, 87)	82 (78, 87)	85 (84, 87)
UC and IBDU vs control	PLS	86 (84, 88)	83 (78, 88)	87 (85, 89)
UC and IBDU vs control	RF	89 (87, 91)	89 (83, 95)	89 (88, 90)
UC and IBDU vs control	LDA	85 (82, 87)	79 (74, 84)	87 (85, 89)
UC and IBDU vs control	SVMR	90 (88, 92)	89 (85, 94)	91 (88, 93)
UC and IBDU vs control	SVML	86 (84, 88)	80 (74, 86)	88 (86, 91)
UC and IBDU vs control	SVMP	85 (83, 86)	76 (71, 81)	88 (86, 90)
CD vs UC	KNN	84 (81, 87)	81 (79, 83)	92 (86, 98)
CD vs UC	PLS	95 (93, 98)	96 (94, 99)	94 (90, 98)
CD vs UC	RF	86 (84, 89)	83 (81, 86)	93 (87, 99)
CD vs UC	LDA	93 (90, 96)	100 (96, 100)	86 (81, 90)
CD vs UC	SVMR	93 (91, 95)	96 (92, 100)	89 (87, 92)
CD vs UC	SVML	82 (79, 84)	82 (79, 86)	81 (76, 86)
CD vs UC	SVMP	95 (93, 98)	96 (93, 99)	94 (90, 99)

Table S5 Summary of CV models for training data

Comparison	Model	Accuracy (95% CI) (%)	Sensitivity (95% CI) (%)	Specificity (95% CI) (%)
IBD vs control	KNN	82 (79, 86)	79 (73, 85)	85 (81, 90)
IBD vs control	PLS	82 (79, 86)	81 (75, 86)	84 (79, 89)
IBD vs control	RF	84 (80, 87)	84 (79, 90)	83 (77, 88)
IBD vs control	LDA	83 (79, 87)	81 (75, 86)	85 (80, 90)
IBD vs control	SVMR	83 (79, 86)	83 (78, 89)	82 (76, 87)
IBD vs control	SVML	86 (83, 89)	81 (76, 86)	91 (87, 95)
IBD vs control	SVMP	84 (80, 88)	82 (77, 87)	85 (80, 90)
CD vs control	KNN	90 (86, 94)	88 (80, 94)	92 (87, 97)
CD vs control	PLS	91 (87, 95)	90 (83, 96)	92 (86, 97)
CD vs control	RF	94 (91, 97)	95 (91, 99)	93 (88, 97)
CD vs control	LDA	90 (85, 94)	88 (81, 95)	91 (85, 97)
CD vs control	SVMR	94 (90, 97)	100 (99, 100)	88 (81, 94)
CD vs control	SVML	94 (90, 97)	92 (87, 97)	95 (90, 99)
CD vs control	SVMP	95 (92, 98)	98 (93, 100)	92 (87, 97)
UC vs control	KNN	86 (82, 89)	55 (42, 66)	97 (94, 99)
UC vs control	PLS	91 (88, 94)	78 (66, 88)	96 (94, 98)
UC vs control	RF	92 (89, 95)	75 (64, 86)	99 (97, 100)
UC vs control	LDA	89 (85, 92)	78 (66, 88)	93 (89, 97)
UC vs control	SVMR	89 (86, 93)	72 (60, 82)	96 (93, 98)
UC vs control	SVML	89 (85, 92)	78 (66, 88)	93 (89, 96)
UC vs control	SVMP	93 (90, 95)	75 (64, 84)	99 (98, 100)
UC and IBDU vs control	KNN	85 (80, 89)	66 (55, 77)	93 (89, 96)
UC and IBDU vs control	PLS	88 (84, 92)	71 (61, 81)	95 (92, 98)
UC and IBDU vs control	RF	90 (86, 93)	70 (59, 79)	99 (97, 100)
UC and IBDU vs control	LDA	85 (81, 89)	70 (59, 80)	92 (87, 95)
UC and IBDU vs control	SVMR	89 (85, 93)	74 (63, 84)	96 (94, 98)
UC and IBDU vs control	SVML	82 (78, 87)	69 (58, 79)	88 (83, 93)
UC and IBDU vs control	SVMP	89 (85, 92)	76 (66, 85)	94 (91, 97)
CD vs UC	KNN	84 (79, 89)	97 (93, 100)	66 (54, 78)
CD vs UC	PLS	96 (92, 99)	99 (97, 100)	91 (84, 98)
CD vs UC	RF	90 (85, 94)	97 (92, 100)	81 (72, 90)
CD vs UC	LDA	92 (88, 96)	89 (83, 95)	97 (92, 100)
CD vs UC	SVMR	94 (90, 97)	96 (91, 100)	92 (84, 98)
CD vs UC	SVML	85 (80, 90)	90 (84, 95)	78 (68, 88)
CD vs UC	SVMP	97 (93, 99)	99 (97, 100)	94 (88, 100)

Table S6 Summary of DCV models for training data

Comparison	Model	Accuracy (95% CI) (%)	Sensitivity (95% CI) (%)	Specificity (95% CI) (%)
IBD vs control	KNN	81 (78, 83)	76 (73, 80)	85 (82, 88)
IBD vs control	PLS	79 (77, 81)	74 (70, 77)	85 (82, 88)
IBD vs control	RF	83 (80, 85)	83 (80, 86)	82 (79, 86)
IBD vs control	LDA	81 (79, 83)	79 (76, 82)	83 (80, 86)
IBD vs control	SVMR	81 (80, 83)	83 (81, 86)	79 (76, 82)
IBD vs control	SVML	81 (79, 83)	78 (74, 81)	85 (82, 88)
IBD vs control	SVMP	82 (80, 84)	82 (79, 85)	82 (79, 86)
CD vs control	KNN	86 (84, 89)	81 (77, 86)	91 (88, 94)
CD vs control	PLS	87 (85, 90)	85 (80, 89)	90 (86, 93)
CD vs control	RF	93 (90, 94)	93 (90, 97)	92 (89, 95)
CD vs control	LDA	85 (82, 87)	82 (78, 86)	87 (83, 91)
CD vs control	SVMR	91 (89, 93)	96 (93, 98)	86 (83, 90)
CD vs control	SVML	90 (88, 92)	89 (85, 93)	91 (88, 94)
CD vs control	SVMP	94 (92, 96)	96 (93, 99)	91 (88, 95)
UC vs control	KNN	86 (85, 88)	59 (52, 65)	96 (95, 98)
UC vs control	PLS	88 (87, 90)	71 (65, 77)	94 (92, 96)
UC vs control	RF	91 (89, 92)	70 (63, 75)	98 (97, 99)
UC vs control	LDA	88 (86, 90)	76 (71, 81)	93 (91, 95)
UC vs control	SVMR	89 (87, 90)	80 (75, 85)	92 (90, 94)
UC vs control	SVML	87 (85, 89)	66 (61, 71)	95 (93, 97)
UC vs control	SVMP	89 (88, 91)	77 (72, 82)	94 (92, 96)
UC and IBDU vs control	KNN	84 (82, 86)	65 (60, 70)	93 (91, 94)
UC and IBDU vs control	PLS	85 (83, 87)	65 (60, 70)	94 (92, 96)
UC and IBDU vs control	RF	87 (85, 89)	65 (60, 71)	97 (96, 98)
UC and IBDU vs control	LDA	83 (81, 85)	68 (63, 74)	89 (87, 92)
UC and IBDU vs control	SVMR	87 (85, 89)	77 (72, 81)	92 (89, 94)
UC and IBDU vs control	SVML	81 (79, 83)	56 (50, 62)	93 (90, 95)
UC and IBDU vs control	SVMP	87 (85, 89)	74 (69, 78)	93 (91, 95)
CD vs UC	KNN	84 (81, 87)	96 (94, 98)	67 (61, 73)
CD vs UC	PLS	93 (91,95)	96 (93, 98)	89 (85, 93)
CD vs UC	RF	87 (85, 90)	95 (92, 97)	77 (71, 83)
CD vs UC	LDA	87 (84, 90)	88 (84, 92)	86 (81, 90)
CD vs UC	SVMR	91 (89, 93)	88 (85, 92)	95 (93, 98)
CD vs UC	SVML	87 (85, 90)	90 (87, 93)	84 (79, 89)
CD vs UC	SVMP	92 (90, 94)	92 (89, 95)	92 (87, 96)

Table S7 Summary of LASSO models for training data

Comparison	Accuracy (95% CI) (%)	Sensitivity (95% CI) (%)	Specificity (95% CI) (%)
IBD vs control	79 (64, 94)	72 (44, 99)	87 (70, 100)
CD vs control	96 (80, 100)	92 (74, 100)	100 (86, 100)
UC vs control	82 (66, 98)	40 (18, 62)	97 (87, 100)
UC and IBDU vs control	79 (63, 95)	44 (26, 62)	95 (83, 100)
CD vs UC	73 (55, 91)	41 (19, 63)	91 (81, 100)

Table S8 Summary of LOOCV models for validation data

Comparison	Model	Accuracy (95% CI) (%)	Sensitivity (95% CI) (%)	Specificity (95% CI) (%)
IBD vs control	KNN	43 (39, 46)	44 (38, 50)	41 (35, 47)
IBD vs control	PLS	57 (54, 61)	57 (52, 62)	57 (51, 63)
IBD vs control	RF	55 (52, 59)	55 (49, 60)	56 (50, 62)
IBD vs control	LDA	59 (55, 62)	59 (53, 64)	59 (53, 66)
IBD vs control	SVMR	45 (42, 48)	45 (38, 52)	44 (35, 53)
IBD vs control	SVML	75 (70, 80)	82 (75, 89)	71 (61, 80)
IBD vs control	SVMP	57 (53, 61)	57 (49, 65)	58 (49, 67)
CD vs control	KNN	63 (58, 67)	63 (56, 69)	63 (56, 69)
CD vs control	PLS	59 (54, 64)	59 (51, 66)	60 (52, 68)
CD vs control	RF	69 (64, 74)	71 (64, 79)	67 (61, 73)
CD vs control	LDA	66 (61, 70)	69 (63, 76)	63 (57, 70)
CD vs control	SVMR	75 (70, 80)	83 (74, 93)	70 (60, 80)
CD vs control	SVML	75 (69, 81)	83 (75, 92)	70 (60, 80)
CD vs control	SVMP	75 (69, 81)	83 (74, 92)	70 (60, 80)
UC vs control	KNN	60 (50,70)	58 (41, 76)	63 (43, 82)
UC vs control	PLS	55 (44, 66)	56 (38, 73)	55 (38, 71)
UC vs control	RF	50 (39, 61)	50 (33, 67)	50 (34, 66)
UC vs control	LDA	55 (43, 67)	56 (38, 73)	55 (36, 73)
UC vs control	SVMR	55 (46, 64)	56 (37, 74)	55 (34, 75)
UC vs control	SVML	50 (38, 62)	50 (32, 68)	50 (32, 68)
UC vs control	SVMP	60 (49, 71)	58 (38, 79)	63 (44, 81)
UC and IBDU vs control	KNN	29 (20, 39)	33 (18, 49)	22 (8, 36)
UC and IBDU vs control	PLS	46 (36, 56)	47 (30, 63)	44 (27, 61)
UC and IBDU vs control	RF	42 (32, 51)	42 (26, 57)	42 (25, 58)
UC and IBDU vs control	LDA	33 (23, 44)	33 (18, 49)	33 (16, 50)
UC and IBDU vs control	SVMR	0	0	0
UC and IBDU vs control	SVML	75 (65, 86)	71 (52, 91)	80 (64, 96)
UC and IBDU vs control	SVMP	17 (8, 26)	17 (0, 36)	17 (0, 34)
CD vs UC	KNN	81 (76, 85)	82 (77, 87)	78 (66, 89)
CD vs UC	PLS	46 (41, 51)	57 (50, 64)	33 (25, 42)
CD vs UC	RF	62 (57, 66)	65 (61, 69)	50 (42, 59)
CD vs UC	LDA	85 (79, 90)	88 (82, 93)	80 (71, 89)
CD vs UC	SVMR	69 (65, 73)	68 (63, 73)	75 (66, 85)
CD vs UC	SVML	73 (69, 77)	80 (75, 85)	64 (54, 74)
CD vs UC	SVMP	73 (69,77)	74 (69, 79)	71 (61, 82)

Table S9 Summary of CV models for validation data

Comparison	Model	Accuracy 95% CI (%)	Sensitivity 95% CI (%)	Specificity 95% CI (%)
IBD vs control	KNN	42 (35, 48)	49 (39, 59)	35 (26, 44)
IBD vs control	PLS	61 (54, 68)	63 (54, 73)	59 (51, 68)
IBD vs control	RF	53 (47, 59)	61 (51, 71)	45 (35, 57)
IBD vs control	LDA	59 (53, 66)	62 (53, 71)	57 (47, 67)
IBD vs control	SVMR	51 (45, 57)	53 (44, 62)	49 (39, 59)
IBD vs control	SVML	71 (65, 77)	62 (52, 71)	81 (73, 88)
IBD vs control	SVMP	61 (55, 67)	63 (54, 73)	59 (51, 69)
CD vs control	KNN	63 (56, 71)	60 (46, 72)	69 (56, 82)
CD vs control	PLS	70 (62, 78)	73 (60, 84)	70 (58, 82)
CD vs control	RF	72 (63, 81)	72 (60, 84)	73 (62, 84)
CD vs control	LDA	67 (58, 76)	63 (48, 76)	73 (60, 84)
CD vs control	SVMR	72 (63, 80)	64 (50, 76)	82 (70, 90)
CD vs control	SVML	74 (66, 82)	68 (56, 80)	82 (72, 92)
CD vs control	SVMP	75 (67, 82)	69 (58, 80)	83 (74, 92)
UC vs control	KNN	66 (54, 76)	70 (56, 84)	61 (44, 76)
UC vs control	PLS	60 (48, 70)	59 (44, 76)	60 (44, 76)
UC vs control	RF	56 (44, 66)	52 (36, 68)	60 (44, 76)
UC vs control	LDA	61 (50, 72)	59 (44, 76)	62 (48, 76)
UC vs control	SVMR	62 (50, 74)	64 (48, 80)	60 (44, 76)
UC vs control	SVML	55 (44, 66)	53 (36, 68)	56 (40, 72)
UC vs control	SVMP	67 (56, 78)	75 (60, 88)	59 (44, 76)
UC and IBDU vs control	KNN	35 (26, 44)	50 (36, 66)	19 (8, 32)
UC and IBDU vs control	PLS	50 (40, 61)	51 (36, 66)	49 (34, 66)
UC and IBDU vs control	RF	42 (32, 51)	41 (26, 58)	44 (28, 60)
UC and IBDU vs control	LDA	40 (31, 50)	40 (24, 56)	40 (26, 54)
UC and IBDU vs control	SVMR	46 (37, 56)	59 (44, 72)	39 (24, 54)
UC and IBDU vs control	SVML	68 (58, 78)	76 (62, 88)	61 (46, 76)
UC and IBDU vs control	SVMP	53 (42, 64)	58(42, 74)	49 (34, 64)
CD vs UC	KNN	79 (70, 87)	87 (76, 96)	67 (52, 84)
CD vs UC	PLS	66 (58, 75)	80 (68, 92)	47 (32, 64)
CD vs UC	RF	70 (61, 78)	79 (68, 88)	58 (40, 72)
CD vs UC	LDA	82 (74, 89)	82 (72, 92)	80 (68, 92)
CD vs UC	SVMR	73 (65, 81)	97 (90, 100)	38 (20, 52)
CD vs UC	SVML	76 (68, 85)	80 (68, 90)	69 (56, 84)
CD vs UC	SVMP	80 (72, 87)	93 (86, 100)	60 (44, 76)

Table S10 Summary of DCV models for validation data

Comparison	Model	Accuracy (95% CI) (%)	Sensitivity (95% CI) (%)	Specificity (95% CI) (%)
IBD vs control	KNN	44 (40,47)	52 (46,58)	36 (31, 42)
IBD vs control	PLS	56 (53, 60)	56 (51, 61)	56 (51,62)
IBD vs control	RF	51 (48, 54)	57 (51, 62)	46 (40, 52)
IBD vs control	LDA	58 (55, 62)	62 (57,67)	55 (48, 62)
IBD vs control	SVMR	47 (44, 50)	45 (38, 52)	49 (40, 58)
IBD vs control	SVML	54 (50, 59)	54 (47, 61)	55 (45, 64)
IBD vs control	SVMP	47 (43, 51)	47 (39, 55)	48 (39, 57)
CD vs control	KNN	62 (58, 66)	58 (52, 65)	66 (60, 73)
CD vs control	PLS	62 (57, 67)	62 (55, 69)	63 (56, 71)
CD vs control	RF	65 (60, 69)	65 (57, 72)	65 (59, 72)
CD vs control	LDA	67 (63, 72)	66 (59,72)	69 (63, 76)
CD vs control	SVMR	50 (44, 55)	46 (37, 56)	50 (40, 60)
CD vs control	SVML	59 (53, 65)	60 (51, 68)	59 (49, 70)
CD vs control	SVMP	59 (53, 64)	54 (44, 62)	62 (52, 72)
UC vs control	KNN	56 (51, 61)	60 (51, 68)	51 (42, 61)
UC vs control	PLS	56 (50, 61)	55 (46, 64)	56 (48, 64)
UC vs control	RF	55 (49, 60)	55 (46, 64)	54 (46, 62)
UC vs control	LDA	49 (44, 56)	51 (43, 60)	48 (39, 57)
UC vs control	SVMR	41 (36, 45)	36 (26, 45)	44 (34, 54)
UC vs control	SVML	48 (42, 54)	47 (38, 56)	49 (39, 58)
UC vs control	SVMP	47 (41, 52)	45 (35, 56)	46 (37, 55)
UC and IBDU vs control	KNN	42 (37, 47)	54 (47, 62)	28 (21, 35)
UC and IBDU vs control	PLS	48 (44, 54)	50 (41, 57)	46 (38, 55)
UC and IBDU vs control	RF	43 (39, 48)	40 (32, 48)	45 (37, 53)
UC and IBDU vs control	LDA	44 (39, 50)	44 (36, 52)	43 (35, 52)
UC and IBDU vs control	SVMR	52 (48, 57)	48 (39, 57)	55 (47, 63)
UC and IBDU vs control	SVML	48 (44, 54)	48 (39, 58)	48 (40, 56)
UC and IBDU vs control	SVMP	47 (43, 52)	49 (40, 59)	43 (34, 52)
CD vs UC	KNN	71 (67, 76)	84 (79, 89)	48 (37, 59)
CD vs UC	PLS	60 (54, 64)	71 (64, 78)	42 (33, 50)
CD vs UC	RF	69 (65, 73)	86 (81, 90)	41 (32, 49)
CD vs UC	LDA	66 (60, 71)	67 (61, 73)	62 (53, 71)
CD vs UC	SVMR	71 (67, 74)	83 (78, 88)	45 (36, 55)
CD vs UC	SVML	70 (65, 74)	82 (77, 87)	44 (33, 53)
CD vs UC	SVMP	71 (67, 75)	86 (80, 90)	43 (33, 53)

Table S11 Summary of LASSO models for validation data

Comparison	Accuracy (95% CI) (%)	Sensitivity (95% CI) (%)	Specificity (95% CI) (%)
IBD vs control	57 (41, 72)	50 (28, 72)	68 (50, 85)
CD vs control	53 (33, 73)	31 (8, 55)	44 (22, 65)
UC vs control	68 (54, 82)	80 (68, 92)	50 (28, 72)
UC and IBDU vs control	60 (42, 78)	58 (45, 72)	42 (20, 64)
CD vs UC	64 (56,72)	61 (57, 75)	70 (54, 86)

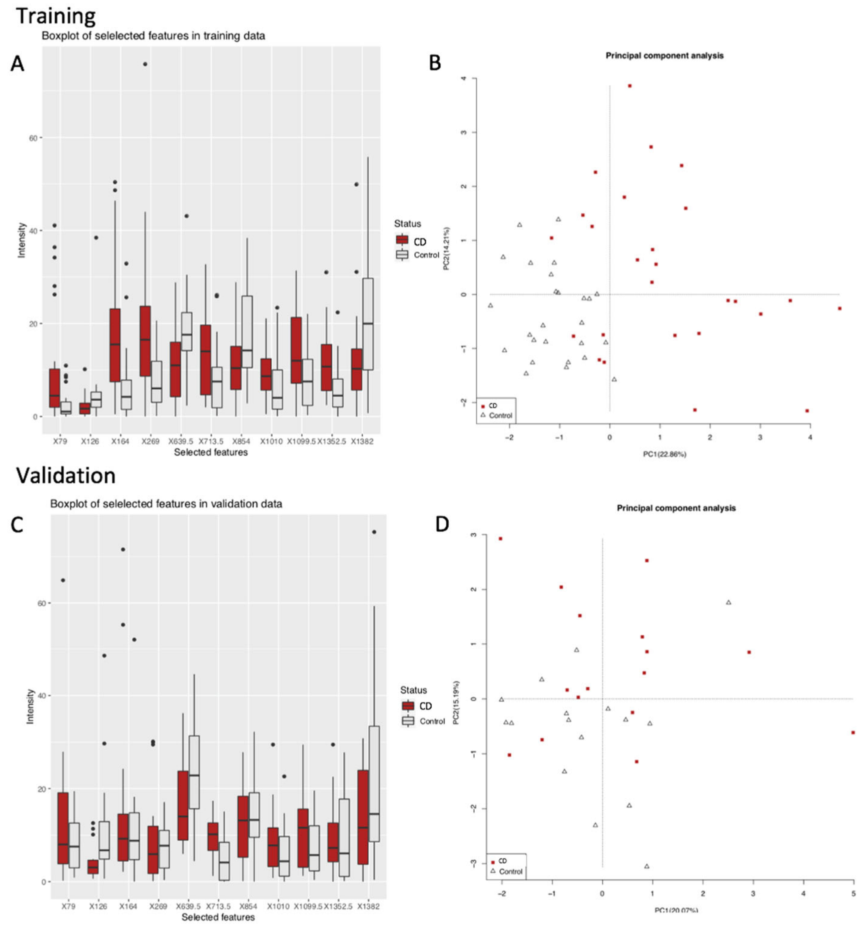

Supplementary Figure 1 Selected training features and validation for CD vs Controls. Boxplots of features selected by the model in the training set (A) and the same features used in the validation (C) and PCAs based on selected features for both training (B) and validation sets (D).

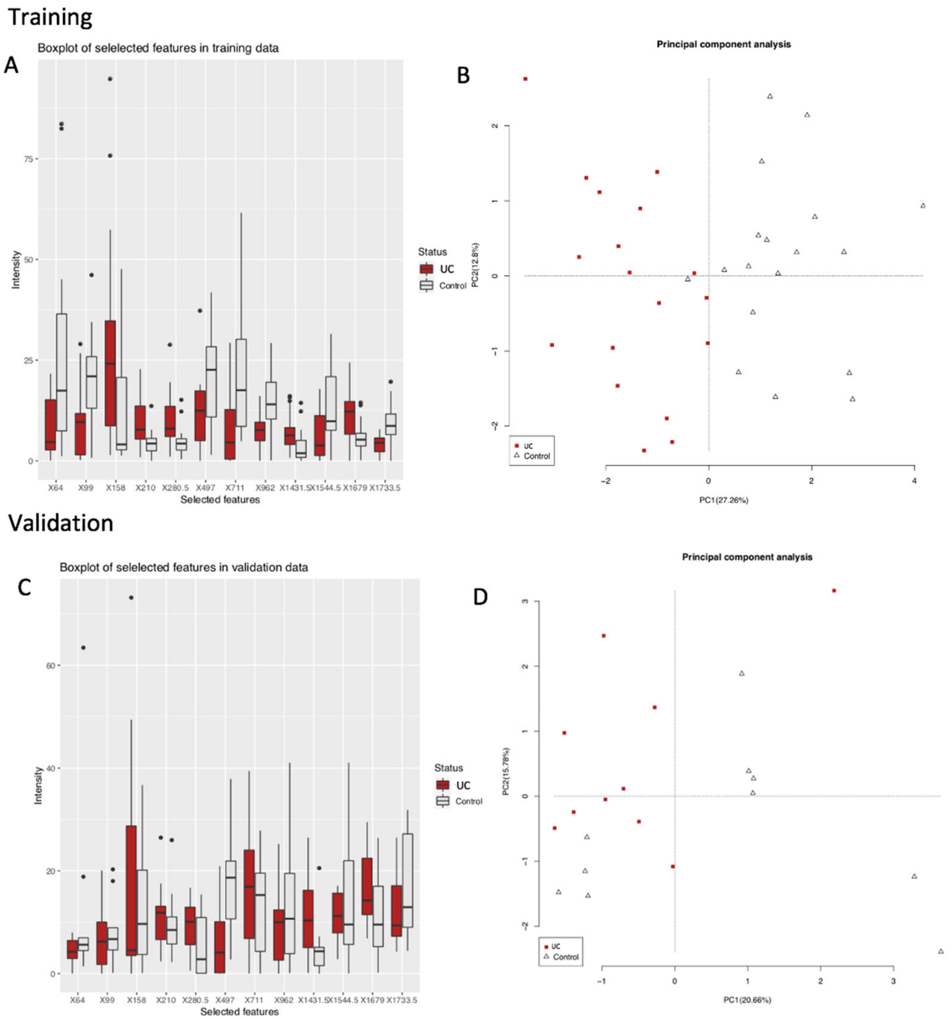

Supplementary Figure 2 Selected training features and validation for UC vs Controls. Boxplots of features selected by the model in the training set (A) and the same features used in the validation (C) and PCAs based on selected features for both training (B) and validation sets (D).

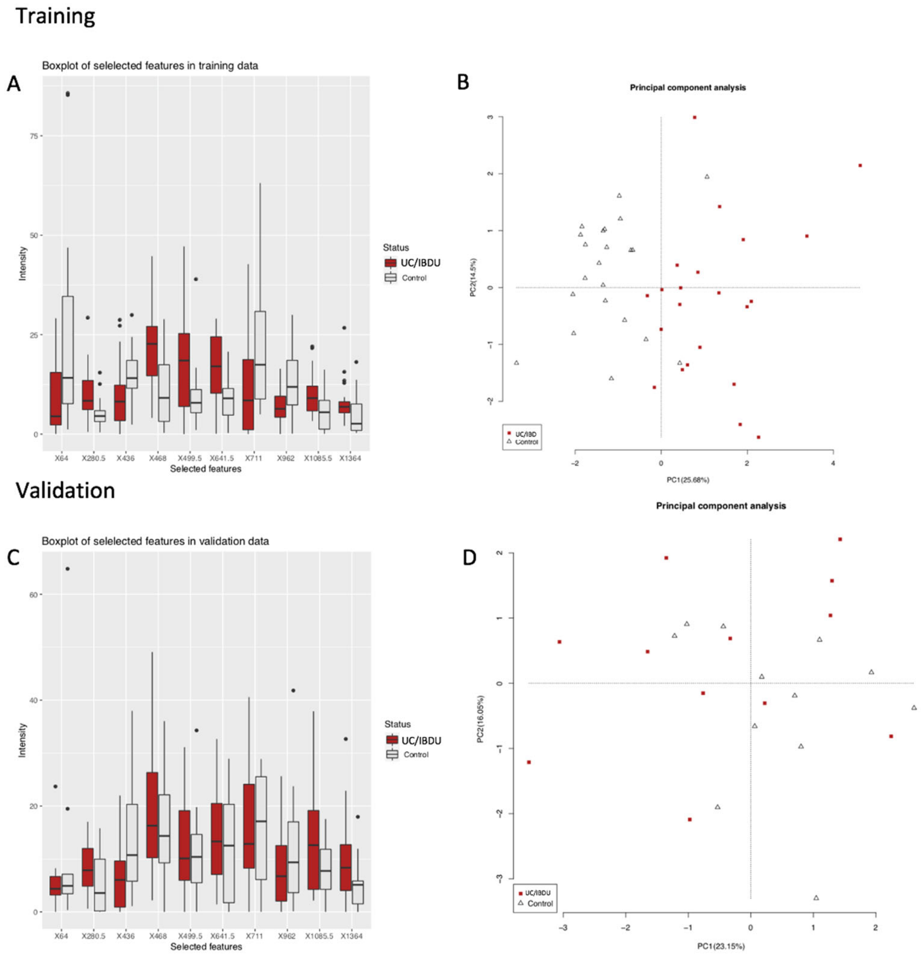

Supplementary Figure 3 Selected training features and validation for UC/IBDU vs Controls. Boxplots of features selected by the model in the training set (A) and the same features used in the validation (C) and PCAs based on selected features for both training (B) and validation sets (D).

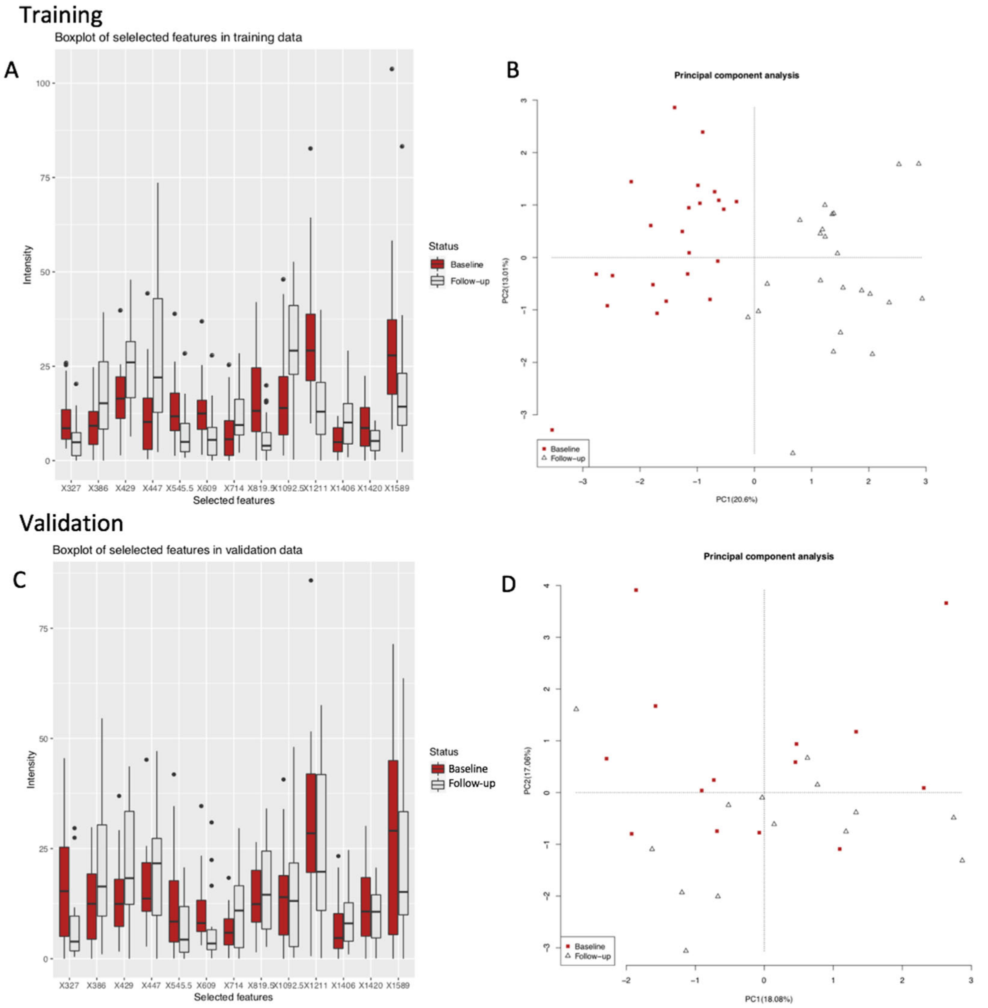

Supplementary Figure 4 Selected training features and validation for baseline IBD vs 3-month FUP. Boxplots of features selected by the model in the training set (A) and the same features used in the validation (C) and PCAs based on selected features for both training (B) and validation sets (D).
